# Pandemic grief in El Salvador: factors that predict dysfunctional grief due to a COVID-19 death among Salvadoran adults

**DOI:** 10.1186/s41155-023-00250-6

**Published:** 2023-03-29

**Authors:** Marlon Elías Lobos-Rivera, Angélica Nohemy Flores-Monterrosa, Jennifer Carolina Tejada-Rodríguez, Edgardo René Chacón-Andrade, Tomás Caycho-Rodríguez, Sherman A. Lee, Pablo D. Valencia, Carlos Carbajal-León, Lindsey W. Vilca, Mario Reyes-Bossio, Miguel Gallegos

**Affiliations:** 1 Universidad Tecnológica de El Salvador, San Salvador, El Salvador; 2 Facultad de Psicología, Universidad Científica del Sur, Lima, Perú; 3 Christopher Newport University, Newport News, USA; 4 Facultad de Estudios Superiores Iztacala, Universidad Nacional Autónoma de Mexico, Tlalnepantla de Baz, State of Mexico Mexico; 5 South American Center for Education and Research in Public Health, Universidad Norbert Wiener, Lima, Perú; 6 Facultad de Psicología, Universidad Peruana de Ciencias Aplicadas, Lima, Perú; 7 Universidad Católica del Maule, Talca, Chile; 8 Universidad Nacional de Rosario, Santa Fe, Argentina; 9 Consejo Nacional de Investigaciones Científicas Y Técnicas, Buenos Aires, Argentina

**Keywords:** Grief, Pandemic, COVID-19, Salvadorans, Anxiety

## Abstract

Thousands of people have died of COVID-19 in El Salvador. However, little is known about the mental health of those who are mourning the loss of a loved one to COVID-19. Therefore, the objective of this study was to examine the dysfunctional grief associated with COVID-19 death among Salvadoran adults. A sample of 435 Salvadorans (*M* = 29 years; SD = 8.75) who lost a family member or loved one to COVID-19 completed a digital survey using the Google Forms platform, during April 2 and 28, 2022. The results revealed that 35.1% reported clinically elevated symptoms of dysfunctional grief and among those mourners, and 25.1% also exhibited clinical levels of coronavirus anxiety. A binary logistic regression revealed that predictor variables such as COVID-19 anxiety (*p* = .003), depression (*p* = .021), and COVID-19 obsession (*p* = .032) were significant (*χ*^2^ = 84.31; Nagelkerke *R*^2^ = .242) and predict a 24.2% chance of dysfunctional bereavement.

## Introduction

The death of a loved one can be experienced by anyone at some point in their lives, resulting in specific physical, emotional, cognitive, and social reactions to the loss (Işıklı et al., 2022). Symptoms of grief include sadness, a feeling of emptiness and meaninglessness in life, mistrust, trouble accepting loss, anger, and confusion of identity (Gesi et al., 2020; Zhai & Du, 2020). While most bereaved people adapt to the death of their loved one over time, about 2 to 10% of people find it harder to adjust to the death, creating difficulties in the grieving process, called complicated grief (Lenferink et al., 2020). Complicated grief is characterized by a prolongation of the normal grieving process and a stagnation in some of the stages of grief and mourning that are specific to it in the approach to grief and pain, which hinder the person’s ability to return to normal psychosocial functioning (Larrotta-Castillo et al., 2020). Evidence suggests that the lack of emotional regulation in complicated grief generates behavioral consequences, such as isolation and fatigue; psychological, such as the presence of depressive symptoms and suicidal ideation; and physical, such as an increased likelihood of heart disease and mortality (Bertuccio & Runion, 2020; Lee & Neimeyer, 2022).

In the case of the COVID-19 pandemic, the percentage of people who experience complications in the grieving process may be higher (Skalski et al., 2022). The COVID-19 pandemic has caused a devastating amount of physical and mental suffering to people around the world (Brooks et al., 2020; Gallegos et al., 2020; Shigemura et al., 2020). Many of those who lost loved ones to this deadly disease have experienced an especially difficult grieving process, referred to as pandemic grief. Pandemic grief is a complicated bereavement experience caused by a COVID-19 death, characterized by symptoms such as death wishes, identity confusion, apathy, difficulty remembering, and meaninglessness (Lee & Neimeyer, 2022). For example, people who have suffered the loss of a loved one as a result of COVID-19 reported suicidal thoughts and even attempts to end their own lives (Halford et al., 2020; Reger et al., 2020; Wand et al., 2020). In Brazil, losing a loved one or friend to COVID-19 was found to amplify the mourner’s already stressful life, particularly if one had a history of mental illness (Joaquim et al., 2021a, 2021b). Independent studies of Americans who are mourning the death of a significant person to COVID-19 revealed that 56.6 to 66.4% of them suffering disabling levels of dysfunctional grief (Breen et al., 2021; Lee & Neimeyer, 2022; Lee et al., 2021). Slightly lower rates were found among mourners in Pakistan at 49.6% (Ashraf et al., 2022) and Peru at 39.3% (Caycho-Rodrıguez et al., 2021a, 2021b). The lowest numbers of mourners suffering dysfunctional levels of COVID-19 grief were found in the South American countries of Brazil and Chile at 7.3% (Caycho-Rodrıguez et al., 2021a, 2021b).

There are many pandemic-related factors that contribute to the elevated levels of grief found among those who lost loved ones to COVID-19 (Molina-Aguilar, 2021; Lee & Neimeyer, 2022; Vázquez-Bandín, 2020b). One factor has been the disruption of funeral rituals. Specifically, many families were not able to provide or attend funeral and burial ceremonies for their deceased loved ones because of the restrictive measurements to try to contain the coronavirus outbreak (Aguiar et al., 2020). For example, in El Salvador, the Ministry of Health designed a protocol for patients who died of COVID-19, that prohibited wake activities, religious acts, funeral rituals, and other funeral arrangements that could involve crowds of people (Ministerio de Salud, 2020). Another factor that has made losing a loved one to COVID-19 emotionally difficult has been the inability to say goodbye and be with their loved ones before they died (Breen, 2020). Restrictions in travel and prohibitions on visiting COVID-19 patients in hospitals were common during the COVID-19 pandemic. The absence of funeral rituals and proper farewells to loved ones has long been known to be risk factors for complications in the grieving process (Mason et al., 2020; Scheinfeld et al., 2021). In fact, recent research has shown that mourners who experienced distress over these kinds of pandemic-related scenarios tend to suffer from dysfunctional grief and significant functional impairments (Lee & Neimeyer, 2022). Unfortunately, it is estimated that when the pandemic is over, the negative mental health consequences will persist for a long time (Fiorillo & Gorwood, 2020; Gallegos et al., 2022).

Despite the growing scientific and clinical interest in pandemic-related grief, there is still much to learn. In a recent study of 10 Latin American countries (i.e., Bolivia, Brazil, Chile, Colombia, Ecuador, El Salvador, Guatemala, Mexico, Paraguay, Peru), El Salvador reported the highest percentage of people with dysfunctional levels of COVID-19-related grief at 14.6% (Caycho-Rodrıguez et al., 2021a, 2021b). Because the number of Salvadoran mourners continues to rise, as the death toll from COVID-19 now numbers in the thousands (Gobierno de El Salvador, 2021), it is vital that scholars and health professionals know more about dysfunctional grief in this Latin American country. Hence, the purpose of this article was to explore potential predictors of pandemic grief in the Salvadoran population while experiencing civil rights restriction and home quarantine measures due to the COVID-19 pandemic. Additionally, the prevalence of pandemic grief symptoms will be examined.

Specifically, it is hypothesized that COVID-19 anxiety, COVID-19 obsession, and depressive symptoms predict pandemic grief. COVID-19 anxiety is a set of somatic symptoms triggered by thoughts or information about COVID-19 (Lee, 2020a), whereas COVID-19 obsession is defined as a series of excessive and repetitive thoughts that people have about COVID-19 (Choi et al., 2022). It has been suggested that excessive levels of anxiety and repetitive thoughts may be dysfunctional (Kalat & Shiota, 2007) and be a central source of distress for many people who experienced the pandemic (Chen et al., 2021; Lee, 2020a). Previous studies have reported significant relationships between pandemic grief and anxiety symptoms during the pandemic (Caycho-Rodriguez et al., 2021a; Lee et al., 2021). It has also been suggested that obsessive thoughts can lead to complicated grief processes (Parkes, 1998). Finally, it has been observed that pandemic grief is related to the presence of depressive symptoms (Caycho-Rodríguez et al., 2021a). Death situations predict the occurrence of depressive symptoms and the possibility of complicated grief for those who have lost a loved one (Carr et al., 2020).

The findings may provide evidence to support treatments that reduce symptoms of COVID-19 anxiety, obsession, and depression and may be effective for better management of pandemic grief. Similarly, the results may be even more important for a Central American country like El Salvador where 33.4% of households were in extreme poverty, 21.1% lacked drinking water service, and 45.8% lacked access to sanitation (Barraza et al., 2020). Also, during the data collection period, there were 5725 people infected with COVID-19 and 4128 cumulative deaths due to COVID-19. However, in this period, severe cases were reduced by 66.2%, and critical cases by 80.0% (Fundaungo, 2022). Also, like many countries, the Salvadoran government carried out a series of actions for epidemiological control, such as home quarantine, creation of quarantine centers, restrictions on public transportation, temporary closure of the economy, and the prohibition of mass activities and on-site classes throughout the country (Alvarado Batres & Méndez Gutiérrez, 2021). Moreover, before registering known cases of COVID-19 in its territory, the Legislative Assembly of El Salvador decreed a State of Exception that restricted three basic freedoms for 15 days: freedom of transit, freedom of assembly, and freedom to change domicile (Sibrián, 2020). During the period of data collection in El Salvador, the inclusion of all residents in the country in the anti-COVID-19 Vaccination Plan, including temporary and permanent foreign residents, was also initiated (Proyecto Mesoamérica, 2022).

## Method

### Design and sample

This exploratory study used a cross-sectional design (Ato et al., 2013) to study the pandemic grief experiences of Salvadorans. The participants were 435 people who had lost a family member or other loved one due to COVID-19. The sociodemographic characteristics of the sample indicate that the participants had an average age of 29 years (SD = 8.75), the majority were young (between 18 and 30 years), female (61.5%), had not been diagnosed with COVID-19, resided in an urban area, did not suffer from a chronic disease, and considered that the probability of contracting COVID-19 was great and that the severity of COVID-19 was highly severe. Table 1 allows us to observe in greater detail the sociodemographic characteristics of the participants.

**Table 1:** Sociodemographic characteristics of the sample (*n* = 435)

Sociodemographic characteristics	Frequency (%)
**Age**	
From 18 to 24 years	154 (35.4)
From 25 to 30 years	131 (30.1)
From 31 to 40 years	97 (22.3)
From 41 to 59 years	53 (12.2)
**Gender**	
Male	165 (37.9)
Female	268 (61.5)
Nonbinary	1 (0.2)
Transgender	1 (0.2)
**Diagnosis of COVID-19**	
Yes	72 (16.6)
No	210 (48.3)
I don’t know, but I think so	118 (27.1)
I don’t know, but I don’t think so	35 (8.0)
**Residence area**	
Urban	338 (77.7)
Rural	97 (22.3)
**Suffer from a chronic illness**	
No	377 (86.7)
Yes	58 (13.3)
**Probability that a person will die from COVID-19**	
Practically nonexistent	9 (2.1)
Very small	40 (9.2)
Small	103 (23.7)
Large	134 (30.8)
Very large	88 (20.2)
Practically 100%	61 (14.0)
**Severity of COVID-19**	
Nothing serious	5 (1.1)
Somewhat severe	22 (5.1)
Severe	87 (20.0)
Very severe	101 (23.2)
Highly severe	220 (50.6)

The inclusion criteria were the following: (1) individuals from El Salvador, (2) adults, (3) who had lost a relative or loved one to COVID-19, (4) able to respond to an online survey, and (5) who gave informed consent to be part of the study. Anyone who did not meet any of the criteria would not be considered for the study. Participants were selected by non-probability snowball sampling (Baltar & Brunet, 2012). Based on this procedure, once the person who met the inclusion criteria was identified, they were asked to share the online survey with other people who had lost a family member or loved one to COVID-19 and who also met the established criteria. The data collection technique was a digital survey using the Google Forms platform, during April 2 and 28, 2022. As mentioned above, during this period, 5725 people were infected with COVID-19 and 4128 cumulative deaths due to COVID-19 were recorded in El Salvador. However, in this period, severe cases were reduced by 66.2% and critical cases by 80.0% (Fundaungo, 2022). During this period, the country also began to include all residents of the country in the anti-COVID-19 Vaccination Plan, including temporary and permanent foreign residents (Proyecto Mesoamérica, 2022).

The online survey was shared via email, WhatsApp, and social networks such as Facebook and Instagram. At the beginning of the online survey, it was mentioned that the information provided was confidential and would not be shared with third parties and that participants had the possibility of withdrawing from the study at any time without having to justify their decision. Before responding to the sociodemographic, pandemic grief, anxiety, depression, obsession, and well-being questions, participants had to provide their informed consent.

### Instruments

#### Sociodemographic variables questionnaire

An ad hoc questionnaire was developed to collect sociodemographic information from the participants, which asked them about their gender, age, diagnosis of COVID-19, residence, if they suffer from a chronic disease, the perception of the probability that a person will die of COVID-19, the severity of COVID-19, and whether they experienced the death of a family member or loved one from COVID-19.

#### Pandemic Grief Scale [PGS] (Lee & Neimeyer, 2022)

It is an instrument that measures dysfunctional grief due to a COVID-19 loss; it consists of 5 items with a time-anchored response scale of 4 response options (0 = not at all, 1 = several days, 2 = more than half of every day, 3 = almost every day). The total score of the PGS is obtained from the sum of the scores of each item, where a higher score would express a higher frequency of dysfunctional grief symptoms. A total score equal to or greater than 7 (87% sensitivity and 71% specificity) would express the presence of dysfunctional grief from a COVID-19 death, suggesting the presence of further assessment or treatment. The scale has been validated in 10 Latin American countries (Caycho-Rodríguez et al., 2021b), demonstrating that it has adequate psychometric properties in Latin American contexts. In the present study, the scale presents excellent reliability coefficients (α = 0.92; ω = 0.92).

#### Coronavirus Anxiety Scale [CAS] (Lee, 2020a)

This 5-item instrument, with a time-anchored response scale ranging from 0 = not at all to 4 = nearly every day in the past 2 weeks, measures dysfunctional anxiety over the coronavirus. The CAS score ranges from 0 to 20, where a higher value expresses a higher frequency of COVID-19 anxiety symptoms. It has been suggested that a cut-off score greater than 9 (90% sensitivity and 85% specificity) would allow categorization between persons with and without COVID-19-related dysfunctional anxiety. This instrument has been validated in multiple countries on all continents; regarding Latin America, the CAS has been validated in 12 Latin American countries (Caycho-Rodríguez et al., 2022) making it suitable for studying coronavirus anxiety in El Salvador. The CAS has excellent reliability coefficients (*α* = 0.91; *ω* = 0.91) for this study.

#### Generalized Anxiety Disorder-2 [GAD-2] (Kroenke et al., 2007)

The GAD-2 is a scale that, as its name indicates, evaluates symptoms related to generalized anxiety disorder. It has 2 items (“feeling nervous, anxious, or on edge” and “unable to stop worrying or unable to control worry”) with a response scale of 4 response options ranging from 0 = not at all to 3 = almost, every day. The total score of the GAD-2 is obtained from the sum of the scores of the two items and ranges from 0 to 6. Higher scores suggest a higher frequency of generalized anxiety symptoms. A cut-off score of 3 (86% sensitivity and 83% specificity) allows the detection of dysfunctional symptoms of generalized anxiety with and without clinical relevance. For this study, the Cronbach’s Alpha coefficient was solid at 0.82.

#### Patient Health Questionnaire [PHQ-2] (Korenke et al., 2003)

This questionnaire briefly measures symptoms of clinical depression and is composed of 2 items (“feeling discouraged, depressed or hopeless” and “little interest or pleasure in doing things”) with 4 response options (0 = not at all to 3 = almost every day). The total score ranges from 0 to 6, where a cut-off score greater than or equal to 3 (sensitivity 87% and specificity 78%) is adequate to identify persons with major depressive disorder (Löwe et al., 2005). In this study, the instrument has high reliability (*α* = 0.89).

#### Obsession with COVID-19 Scale [OCS] (Lee, 2020b)

The OCS is an instrument that measures the severity of a person’s obsession with COVID-19. This instrument has been translated and adapted into Spanish (Caycho-Rodríguez et al., 2021c) and consists of 4 items with a response scale of 5 options. The instrument has adequate psychometric properties of validity and reliability. In the present study, the instrument exhibited strong reliability (*α* = 0.88; *ω* = 0.89).

### Statistical analysis

First, a descriptive analysis was performed for the Pandemic Grief Scale using frequencies and percentages of each of the items. Second, the statistical descriptions of the presence and absence of pandemic grief and dysfunctional anxiety are examined according to sociodemographics. Third, a Kruskal–Wallis *H* technique (Núñez-Colín, 2018) was applied to check if there were statistical differences based on each sociodemographic characteristic. Last, a Binary logistic regression analysis (Vilà-Baños et al., 2019) was implemented using the intro method to determine which variables predict dysfunctional grief due to COVID-19 death. It should be noted that in the present study, there were no missing data and no participants were excluded.

## Results

**Fig. 1:**
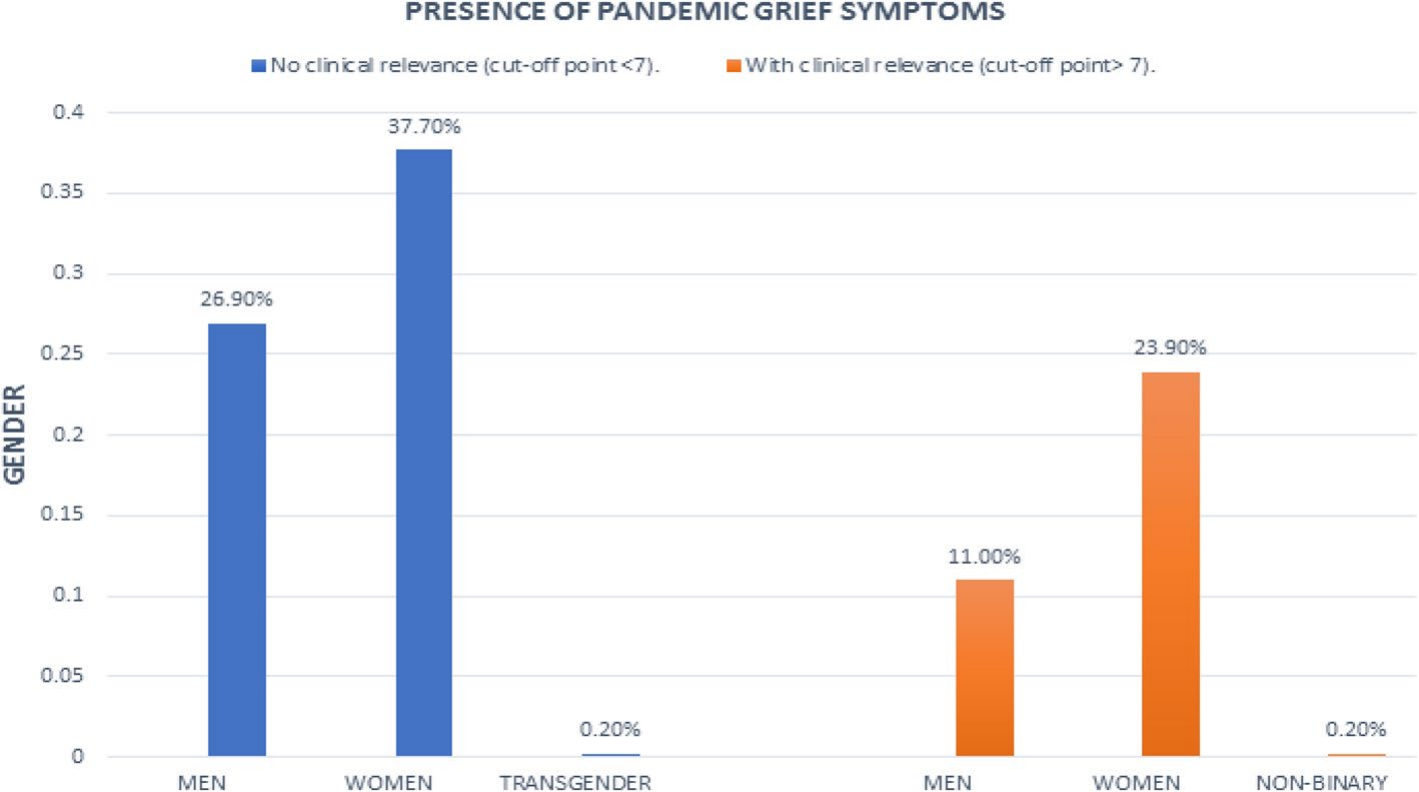
Presence of pandemic grief symptoms with and without clinical relevance in the sample

**Fig. 2:**
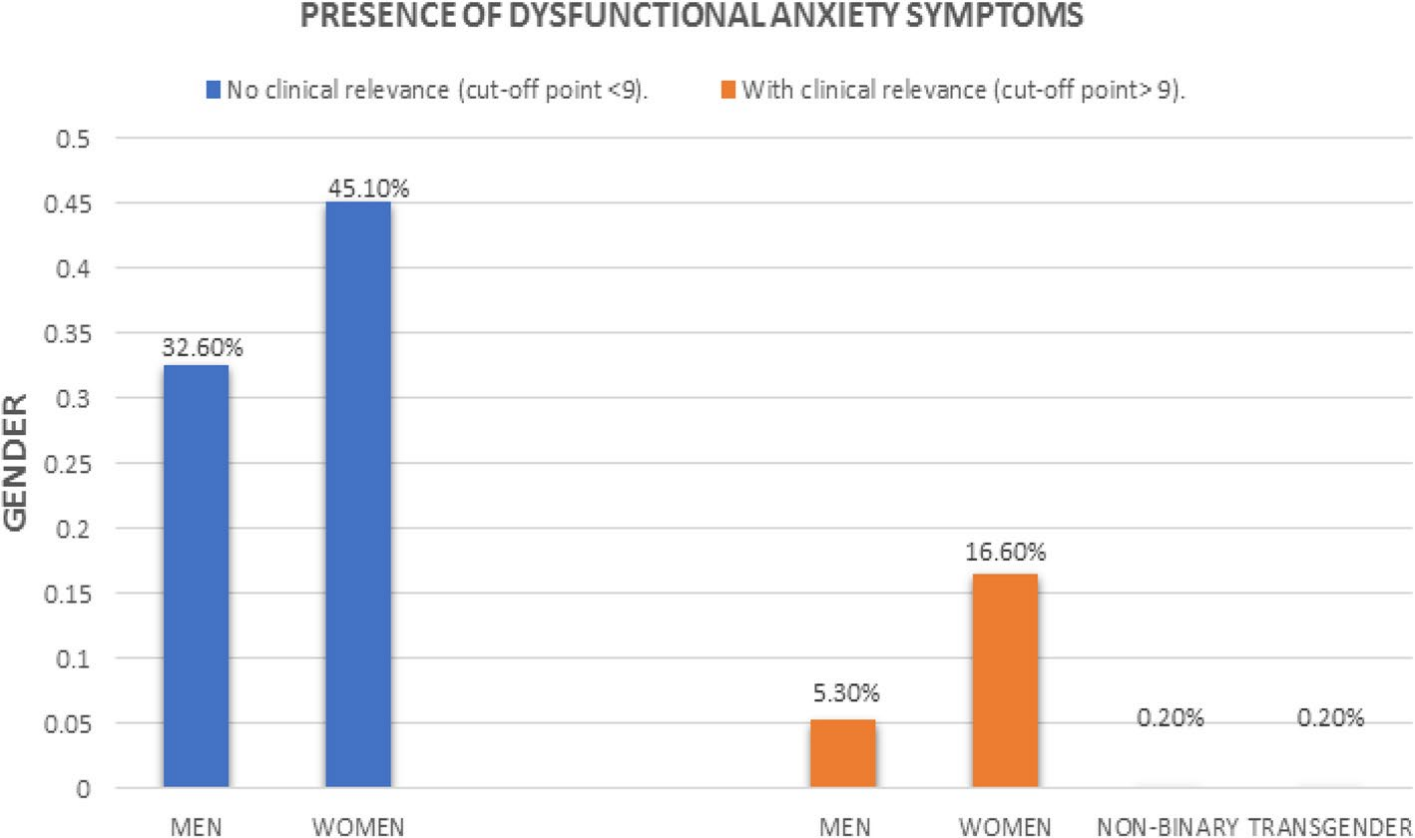
Presence of dysfunctional anxiety symptoms with and without clinical relevance in the sample

Frequencies and percentages of item responses for the Pandemic Grief Scale are presented in Table 2. The results reveal that 18.4% of the participants wanted to die to be with the deceased person, 32.9% experienced confusion about their role in life due to the loss, and 33.1% stated that nothing seemed to matter to them due to their loss, while 29.4% find it difficult to have positive memories of the deceased person. Finally, 27.6% believed that without the deceased person life was meaningless or could not continue. The above percentages were obtained from the sum of the percentages of the responses “Several days,” “More than half of the days,” and “Almost every day.” In terms of clinical levels of psychological dysfunction (Lee & Neimeyer, 2022; Lee, 2020a, 2020b), 153 Salvadorans 35.1% presented symptoms of clinically dysfunctional pandemic grief (see Fig. 1) and 25.1% exhibited clinically relevant symptoms of coronavirus-related anxiety (see Fig. 2).

**Table 2:** Descriptive analysis of the Pandemic Grief Scale items

Ítems	Not at all	Several days	More than half the days	Nearly everyday
1. I wished to die in order to be with the deceased	355 (81.6%)	35 (8.0%)	26 (6.0%)	19 (4.4%)
2. I experienced confusion over my role in life or felt like my identity was diminished because of the loss	292 (67.1%)	70 (16.1%)	42 (9.7%)	31 (7.1%)
3. Nothing seemed to matter much to me because of this loss	291 (66.9%)	74 (17.0%)	39 (9.0%)	31 (7.1%)
4. I found it difficult to have positive memories about the deceased	307 (70.6%)	64 (14.7%)	36 (8.3%)	28 (6.4%)
5. I believed that without the deceased, life was either meaningless, empty, or could not go on	315 (72.4%)	65 (14.9%)	26 (6.0%)	29 (6.7%)

Table 3 presents the descriptive statistics of the dimensions of each instrument. It was found that the standard deviations are close to the mean for most of the variables. In the case of COVID-19 anxiety, the standard deviation is higher than the mean. Skewness and kurtosis remain in the range of − 1.5 and + 1.5, with the exception of pandemic grief, which presents these indicators elevated to the acceptable range. In addition, the Kolmogorov–Smirnov test was applied to each dimension and it was found that they present an asymmetric behavior (*p*-value < 0.001), which gives the guideline to use nonparametric tests in the inferential analysis.

**Table 3:** Descriptive statistics of the dimensions of the measuring instruments

Dimensión	Mean	Standard deviation	Skewness	Kurtosis	K-S test	*p*-values
Pandemic grief	7.44	3.84	1.77	2.24	0.294	< .001
COVID-19 anxiety	4.88	5.32	1.12	0.33	0.180	< .001
Anxiety	2.05	1.95	0.62	− 0.78	0.179	< .001
Depression	2.03	2.00	0.63	− 0.85	0.192	< .001
Obsession with COVID-19	12.13	9.71	0.73	− 0.43	0.127	< .001

Differences in pandemic grief symptoms were found among the sociodemographic variables. Specifically, symptoms of pandemic grief showed statistically significant differences in terms of age (*H* = 18.47; *p*-value = 0.001; Ɛ^2^ = 0.043) and the presence of a chronic disease (*H* = 12.45; *p*-value = 0.001; *Ɛ*^2^ = 0.028). More precisely, people between 18 and 24 years old, followed by those between 41 and 59 years old and those who suffer from a chronic disease, showed the highest levels of dysfunctional grief due to COVID-19 loss (see Table 4). However, the effect size for this finding is small.

**Table 4:** Statistical differences in pandemic grief based on the sociodemographic variables of the sample (*n* = 435)

Sociodemographic variables	*n*	Range	H	*p*-values	Ɛ^2^
**Age**					
From 18 to 24 years	154	239.92	18.47	.001	.043
From 25 to 30 years	131	217.56			
From 31 to 40 years	97	176.08			
From 41 to 59 years	53	232.11			
**Gender**					
Male	165	203.81	3.40	.065	.008
Female	268	225.12			
**Diagnosis of COVID-19**					
Yes	72	205.60	6.40	.094	.015
No	210	208.30			
I don’t know, but I think so	118	235.43			
I don’t know, but I don’t think so	35	242.91			
**Residence area (*n* = 435)**					
Urban	338	218.29	0.01	.992	.000
Rural	97	216.97			
**Suffer from a chronic illness**					
No	377	210.20	12.45	.001	.028
Yes	58	268.68			
**Probability that a person will die from COVID-19**					
Very small	40	188.10	8.96	.062	.021
Small	103	208.43			
Large	134	209.34			
Very large	88	211.03			
Practically 100%	61	251.40			
**Severity of COVID-19**					
Somewhat severe	22	196.00	4.59	.204	.011
Severe	87	201.06			
Very severe	101	207.27			
Highly severe	220	226.94			

Finally, binary logistic regression was performed to identify significant predictors of pandemic grief. The predictor variables for this analysis were COVID-19 anxiety, COVID-19 obsession, depressive symptoms, and generalized anxiety. Preliminary analysis revealed that all significance indices (*p*) were adequate, except for generalized anxiety, which was eliminated because the *p*-value was non-significant (*p* = 0.309). The main results of the analysis revealed that predictor variables such as COVID-19 anxiety (*p* = 0.003; Exp (B) = 1.095 [95% C.I. = 1.031; 1.162]), depression (*p* = 0.021; Exp (B) = 1.170 [95% C.I. = 1.024; 1.336]), and COVID-19 obsession (*p* = 0.032; Exp (B) = 1.036 [95% C.I. = 1.003; 1.070]) were significant (*χ*^2^ = 84.31; Nagelkerke *R*^2^ = 0.242) and predict a 24.2% chance of dysfunctional bereavement. Of all the variables, the one that has the greatest strength in explaining pandemic grief is depression, since its exponential from Exp(B) moves away by more than 1 (see Table 5).

**Table 5:** Binary logistic regression predicting COVID-19 pandemic grief

Model	*χ*^2^ (gl)	Nagelkerke *R*^2^	B	Standard error	*z*	*p*-values	Exp (B)
	84.31 (3)	.242				.001	
Anx. C-19			.091	.031	8.736	.003	1.095
Depr			.157	.068	5.323	.021	1.170
Obs. C-19			.035	.017	4.580	.032	1.036
Constant			− 1.880	.202	86.971	< .001	.153

*Anx.C-19* COVID-19 anxiety, *Depr*. depression, *Obs. C-19* obsession with COVID-19

## Discussion

The loss of a loved one to COVID-19 has been particularly challenging for the bereaved living through this global health crisis (Breen, 2020; Goveas & Shear, 2020; Hamid & Jahangir, 2020). The current study sought to add to our understanding of this unique form of loss, by examining the dysfunctional grief symptoms of Salvadorans who lost loved ones to COVID-19. The first set of results for this study revealed that 35.1% of Salvadorans presented symptoms of clinically dysfunctional grief over a COVID-19 death. Although this prevalence rate is lower than what has been found in American samples (56.6% to 66.4%; Breen et al., 2021; Lee & Neimeyer, 2022; Lee et al., 2021), they are in line with those reported outside of the USA, in such places as Pakistan (49.6%; Ashraf et al., 2022) and Peru (39.3%; Caycho-Rodrıguez et al., 2021a, 2021b). The item analysis provided deeper insight into these pandemic grief experiences by showing that a sizable minority of bereaved Salvadorans experienced dysfunctional grief symptoms. Apathy was the most commonly reported pandemic grief symptom at 33.1% and suicidal ideation was the least frequently endorsed grief symptom at 18.4%. The results also revealed that 25.1% of the sample exhibited clinically relevant symptoms of coronavirus-related anxiety. This rate of coronavirus-related anxiety is similar to what has been reported in the USA (31.2%; Lee et al., 2020) and Mexico (30.3%; Mora-Magana et al., 2022). These findings highlight the plight of many of bereaved Salvadorans who are suffering from the disabling effects of both dysfunctional grief and anxiety.

The results of the sociodemographic analysis also provided additional data regarding Salvadorans bereaved by a COVID-19 death. The findings showed that for this group of bereaved Salvadorans, pandemic grief differed by age and health status. Specifically, the highest levels of dysfunctional grief symptoms were found among young adults (i.e., adults between 18 and 24 years of age) and those who suffer from a chronic disease. Because young adults tend to have relatively fewer experiences with loss and are less emotionally mature than their older counterparts, this finding is understandable. However, the reasons why chronic disease was associated with high levels of dysfunctional grief may be more complicated. Perhaps, those with chronic disease express greater grief than others because they can more closely empathize with the deceased due to their personal experiences with being sick and also being vulnerable to COVID-19. Future research should examine the possibility that empathy is an explanatory variable in this finding. Because bereavement is a known risk factor for health complications (Schulz et al., 2006), particularly heart problems (Llavina-Rubio, 2014), future investigations should also seek to examine if those bereaved by a COVID-19 death and who suffer from a chronic illness become prone to more serious illnesses during the course of their bereavement.

Arguably, the most interesting results came from the regression analysis, which revealed that coronavirus anxiety, COVID-19 obsession, and depressive symptoms were predictors of pandemic grief, accounting for 24.2% of the variance in pandemic grief symptoms. The finding that pandemic grief was positively correlated with a wide range of psychological problems was also reported in several independent studies from Turkey (Evren et al., 2022), the USA (Breen et al., 2021), Peru (Caycho-Rodriguez et al., 2021a, 2021b), and Poland (Skalski et al., 2022). In the case of anxiety about COVID-19 and obsession about COVID-19, the finding is explanatory, as the death of a loved one can generate symptoms of anxiety and recurrent thoughts that, in turn, lead a person to experience a more prolonged mourning, even more so, when the death has been difficult, as when dealing with COVID-19 (Milman et al., 2020; Shear et al., 2012; Zisook et al., 1990). Given that anxiety is one of the most commonly identified disorders in the Salvadoran population (Chacón-Andrade et al., 2020; Gutiérrez-Quintanilla et al., 2020; Lobos-Rivera et al., 2022), the connection between pandemic grief and coronavirus anxiety makes sense. Regarding the relationships between pandemic grief and depressive symptoms, they can be explained due to the restrictions in social interaction that were present. In these circumstances, the elimination of social support made it either impossible, or limited the performance of funeral ceremonies, causing people to experience their grief alone (Mortazavi et al., 2020). These findings would suggest that treatments to reduce symptoms of COVID-19 anxiety, illness obsession, and depression may be effective in improving bereavement management (Marques et al., 2013).

A couple of limitations must be considered when interpreting the findings of this study. First, this study was constrained by its online self-report survey methodology. Future research would benefit from the additional use of clinical interviews, which should be carried out by trained clinical psychologists and psychiatrists who can provide deeper insights into the psychological make-up of the study participants. Second, other important areas of life that have been adversely impacted by the COVID-19 pandemic, such as loss of employment and disrupted routines, were not examined in this study. Thus, future research should incorporate questions regarding these kinds of life-altering impacts and their relation to pandemic grief. Third, the use of online convenience sampling would not allow generalizing the results and would generate an overrepresentation of women and young adults in the sample. This generates a method bias in the findings. Fourth, the use of a cross-sectional design limits testing a prospective prediction and the bidirectional relationships between the variables included in the regression model could not be known with certainty. Fifth, although all participants experienced the death of a family member or loved one, no information was obtained on the specific relationship of the participants to the deceased or the number of loved ones the participants lost.

## Conclusion

Notwithstanding these limitations, this study’s findings are relevant for mental health clinicians and researchers, on a national and international level, who are working with people who are mourning the death of a significant person to COVID-19. In conclusion, a binary logistic regression revealed that predictor variables such as COVID-19 anxiety, depression, and COVID-19 obsession were significant and predict a 24.2% chance of dysfunctional bereavement.

As future lines of investigation, the results of this study suggest that mental health providers working with the bereaved may need to develop intervention plans that consider a wide range of psychological problems, such as coronavirus-related anxiety, in addition to pandemic grief. Also, the presence of a relationship between the practice of mourning rituals and grief reactions during the COVID-19 pandemic has been suggested (Şimşek Arslan, & Buldukoğlu, 2021). Therefore, future studies can use in-depth interviews to assess the impact of mourning rituals on the grieving process during the COVID-19 pandemic. Similarly, it would be important to conduct studies with longitudinal designs with people who have had the death of a family member or loved one during the COVID-19 pandemic. This, taking into consideration that death during the pandemic can generate long-term dysfunctional grief. Finally, incorporating information on the relationship between the bereaved and the deceased should be considered in future studies. This would help to understand whether the grief trajectory is similar or not depending on the closeness or not between the bereaved and the deceased.

## Data Availability

All data related to this study are available from the authors upon request. The data are not yet publicly available because the project group is still processing it.

## Abbreviations

PGS: Pandemic Grief Scale
CAS: Coronavirus Anxiety Scale
GAD-7: Generalized Anxiety Disorder-7
PHQ-2: Patient Health Questionnaire
OCS: Obsession with COVID-19 Scale
